# Functional organization and its implication in evolution of the human protein-protein interaction network

**DOI:** 10.1186/1471-2164-13-150

**Published:** 2012-04-24

**Authors:** Yiqiang Zhao, Sean D Mooney

**Affiliations:** 1 Buck Institute for Research on Aging, Novato, California, USA; 2 Department of Medical and Molecular Genetics, Indiana University School of Medicine, Indianapolis, Indiana, USA

## Abstract

**Background:**

Based on the distinguishing properties of protein-protein interaction networks such as power-law degree distribution and modularity structure, several stochastic models for the evolution of these networks have been purposed, motivated by the idea that a validated model should reproduce similar topological properties of the empirical network. However, being able to capture topological properties does not necessarily mean it correctly reproduces how networks emerge and evolve. More importantly, there is already evidence suggesting functional organization and significance of these networks. The current stochastic models of evolution, however, grow the network without consideration for biological function and natural selection.

**Results:**

To test whether protein interaction networks are functionally organized and their impacts on the evolution of these networks, we analyzed their evolution at both the topological and functional level. We find that the human network is shown to be functionally organized, and its function evolves with the topological properties of the network. Our analysis suggests that function most likely affects local modularity of the network. Consistently, we further found that the topological unit is also the functional unit of the network.

**Conclusion:**

We have demonstrated functional organization of a protein interaction network. Given our observations, we suggest that its significance should not be overlooked when studying network evolution.

## Background

Proteins physically interact with each other in physiological conditions. Individual protein interactions can be direct physical binding or membership within a multiprotein complex, and can be either permanent or transient [1]. It is believed that the diversity of protein-protein interactions (PPI) contribute to the genetic complexity of organisms [2,3]. Thanks to the development of high throughput technology, human PPI data has been greatly accumulated, which provides an opportunity to study that network systematically.

One important question to ask is, "How did the human PPI network emerge and evolve?" Given that the most significant property of the network is that the degree distribution follows a power law [4], several evolutionary models have been proposed to account for this attribute. These include the preferential attachment model, which asserts that a new protein is more likely to interact with well-connected nodes [5,6], and the duplication-divergence model, which emulates gene duplication and the subsequent loss of inherited interactions [7-9]. Both models successfully reproduce the power law degree distribution. Researchers, however, found that the exponent of the degree distribution generated by the preferential attachment model is higher than that from the empirical network [10] and, more importantly, the preferential attachment model fails to reproduce the modularity structure that is observed in most biological networks [11]. Alternatively, the proposed duplication-divergence model is more biologically motivated. With proper parameters, it can reproduce both the power law degree distribution and the modularity structure (through interaction rewiring and/or homomeric duplication, that is, duplication of self-interacting nodes); hence it receives extensive attention as a better candidate mechanism [12,13]. Except studying pure topology, Kim [14] recently found that proteins of close age tend to interact with each other in yeast and proposed a new stochastic model which grows the network analogous to the process of growing protein crystals in solution. The authors claimed that the new model better explains many features of PPI networks. Although increasing features of empirical PPI networks had been captured by current models, all these stochastic models proposed do not require the intervention of natural selection to reproduce the intended topology, nor does it use biological function as a parameter.

On the other hand, people have realized that network structure is relevant for biological function [15,16]. Many efforts have been made to find a relationship between network topology and functional and/or evolutionary properties. It has been reported that interacting proteins tend to be co-evolving [17], co-functional [18] and co-expressed [19,20]. Highly interacting nodes in the network are generally more evolutionarily conserved [21] and tend to be essential and disease causing [4,22]. Based on this information, the PPI network has been successfully used for predicting or prioritizing candidate genes of interest [23-26]. Given that, however, systematic functional analysis of PPI networks is still lacking. Using different datasets and techniques, Yook and Pandey both found correlation between the functional roles and topological structure, indicating that PPI networks are functionally organized [27,28]. In a separate study, by comparing changes of interaction degree in functional classes and the time of origin of proteins, as well as functional heterogeneity at the time of origin, Kunin suggested that functional evolution might be the underlying reason for observed PPI network topological evolution [29]. That study, however did not show in detail how function evolves, nor its relationship with the evolution of network topologies. In opposition to these findings, Wang *et al.*, by breaking down a PPI network into structure modules, found that the network is not functionally organized at the modular level and suggested it evolves neutrally [30]. Whether a PPI network is functionally organized and whether that organization's implication in the evolution of PPI networks is currently inconclusive.

Because functionality is an important aspect of molecular evolution, it is important to clearly address this question in order to have a better understanding of how the PPI network evolves. In this paper, we examine the evolution of a PPI network by dividing human proteins into temporal groups using known phylogenetic information. After doing this, we were able to track the evolutionary changes of the human PPI network at both the topological and functional level. We show the human PPI network functionally organized. In addition, we find that the topological and functional evolution of the human PPI network are not independent of each other. Function affects network topology during evolution, especially on local modularity. This is further supported by the finding that the topological unit is also the functional unit of the human PPI network. Based on our observations, we suggest that an extended model be developed that considers functional significance.

## Results

### Topological evolution of the PPI network

**Figure 1:**
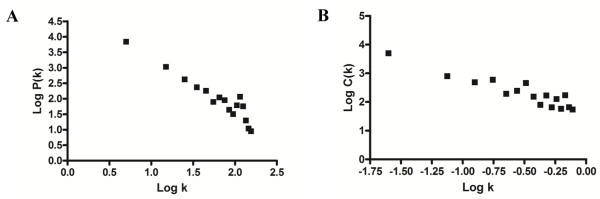
**Distribution of the interaction degree and clustering coefficient of the human PPI network, in a log-log scale**. A. Interaction degree. B. Clustering coefficient.

In our integrated network, there are 9,530 nodes and 65,213 edges. Consistent with previous reports, interaction degree distribution (Figure 1A) exhibits power law behavior [4]. Eighty percent of the nodes interact with fewer than 15 proteins. A small portion of proteins are highly connected, for example, eight nodes are observed to interact with more than 200 proteins. The network also shows hierarchical modularity [31] as reflected in the power law behavior of the clustering coefficient, which is by definition a measure of the degree to which nodes in a graph tend to cluster together (Figure 1B).

**Figure 2:**
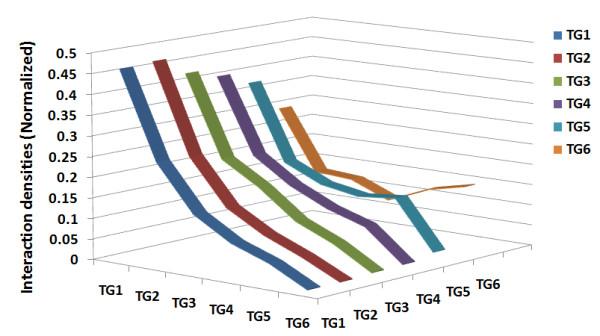
**Interaction densities across temporal groups**. The interaction densities were calculated as described in the Methods. Interaction densities were further normalized according to the overall density for each temporal group (TG) for the purpose of comparison.

In many cases analyses and models of the evolution of PPI networks focus on the evolution of network topology. In order to trace evolutionary changes of the network, human genes were divided into six temporal groups (hereafter referred to as TG; TG1 is the oldest and TG6 is the newest, see Methods). Proteins in older TGs in the network were found to have significantly higher interactions (Kruskal-Wallis test, H = 460.41; df = 5; P < 0.0001), with a progressive decay of interaction degree toward proteins in newer TGs. Proteins in TG1 have the highest degree with an average of 29.5, while those in TG6 have the lowest degree with an average of 3.4 (Mann-Whitney test, P < 0.0001 in both cases; Bonferroni-corrected, see Table 1). However, this is not simply because interactions are created equally, *i.e.*, each node has the same probability of being linked to any other protein and old proteins have had more time to accumulate interactions. If this were the case, we would expect the degree distribution would follow an exponential distribution with a short tail. We also expect a high correlation between interaction degree and protein age, however, the observed correlation is relatively low (Spearman rho = -0.212). The degree of nodes in the network provides only limited information; when both interacting partners are considered, the interaction pattern could be better described with a richer measure. Thus, in more detail, we computed the interaction density (see Methods) across all TGs. The interaction densities were further normalized according to the overall density for each TG (For interaction densities before normalization, see Additional file 1, Table S1). As shown in Figure 2, the higher degree of proteins in older TGs is due to the finding that proteins in the network are more likely to interact with older proteins, regardless of the TG of origin.

**Table 1:** Properties of the PPI network for each temporal group

Temporal group	Approximate group age (MYA)	Gene number in the genome	Gene number in the interaction network	Average interaction degree	Average clustering coefficient	Average Omega
1	990	720	544	29.509	0.212	0.034
2	450	5211	3546	17.399	0.196	0.089
3	360	1929	1093	13.100	0.193	0.096
4	310	2694	1348	10.061	0.180	0.128
5	90	6663	2794	8.623	0.169	0.179
6	50*	1313	205	3.434	0.176	0.168

MYA: million years ago

* Could not be accurately estimated because of our method of assigning temporal groups

Topological modularity is another distinct property of a biological network. Compared to the Erdos-Renyi random network [32], our PPI network shows a significantly higher average clustering coefficient (1000 permutations, P < 0.001). We also found that proteins from older TGs were, in general, more clustered when comparing clustering coefficients among TGs (Kruskal-Wallis test, H = 118.49; df = 5; P < 0.0001, Table 1). Besides examining direct interactions, we also examined the network distance (defined as shortest path length between nodes), which measures indirect connections for protein pairs in the network. As shown in Table 2, proteins belonging to older TGs were more closely connected to other proteins in the network (*i.e.*, the minimum distance between them is less). Conversely, newer proteins were less connected to others in the network. On average, proteins from TG1 to TG6 need 3.877, 4.026, 4.087, 4.176, 4.271 and 4.412 steps, respectively, to reach other proteins in the network (Kruskal-Wallis test, H = 97.92; df = 5; P < 0.0001). Particularly, proteins from TG1 are closest to other proteins from TG1 with an average step of 3.611, while proteins from TG6 are farthest from other proteins from TG6 with an average step of 4.655.

**Table 2:** Average network distances across temporal groups

	TG1	TG2	TG3	TG4	TG5	TG6
TG1	3.611	3.771	3.835	3.931	4.035	4.183
TG2		3.922	3.986	4.078	4.181	4.325
TG3			4.044	4.138	4.239	4.387
TG4				4.227	4.322	4.463
TG5					4.404	4.538
TG6						4.655

The network distance is defined as shortest path length between a pair of nodes

Distances are averaged according to the temporal group combinations to which each pair of nodes belongs

TG: Temporal group

**Figure 3:**
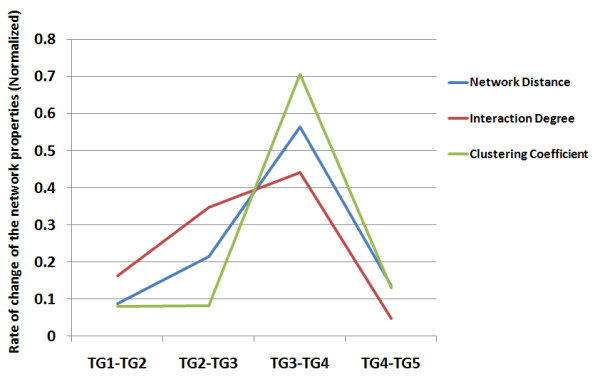
**Rate of change of network properties in different evolutionary stages**. Rate of change of network properties in TG5-TG6 was not done because it could not be accurately estimated. Rate of change of network properties was normalized according to the overall changes for each network property for the purpose of comparison. (For rate of change of the network properties before normalization, see Additional file 1, Table S2.) TG: Temporal group.

Since network topologies vary among TGs, a question to ask is: "Is the change constant, or if not, in which evolutionary stage was the network topology changing the fastest?" For each temporal group, an approximate age (millions of years ago, Mya) is obtained based on previous molecular phylogenetic studies [33]. The rates of topological changes were measured by the differences in topological properties per unit of time. Under the neutral model without any functional significance, a constant rate of topological changes is expected. It is found, however, that during the stage from TG3 to TG4 the rate change in interaction degree, clustering coefficient and network distance were up to 10 times faster than other stages (Figure 3). By checking the major evolutionary events in this period, it suggests that TG3 to TG4 represents the evolution from cold-blooded animals to warm-blooded animals.

In summary, tracking the topological evolution of a PPI network by dividing proteins in the network into six temporal groups showed that more ancient proteins were more highly connected to other proteins in the PPI network. We also showed the topological changes during evolution were not uniform; they accelerated during the stage of evolution from cold-blooded animals to warm-blooded animals.

### Functional organization and evolution of the PPI network

It is obvious that ancient genes that are not lost are conserved. By calculating omega (Ka/Ks) between human and mouse orthologs (see Methods), genes from the older TGs do show lower omega values, indicating stronger selective conservation (Kruskal-Wallis test, H = 2682.06; df = 5; P < 0.0001, Table 1). Evolutionary conservation is often interpreted as having functional importance. Our observations, as described above, together with previous reports on the association between network properties and functional or evolutionary properties, make us wonder whether the network is functionally organized, and, furthermore, whether topological evolution of the network is associated with functional evolution.

To test whether and how a PPI network is functionally organized, we performed functional enrichment tests for the proteins within individual TGs. We find that functional categories, such as "cell cycle", "protein and nucleic acid metabolism" in biological process and "nucleic acid binding" in molecular function can be repeatedly detected across most TGs, while some other functions are specifically enriched for proteins from particular TGs such as "sensory perception" and "blood circulation and gas exchange", suggesting both areas of functional conservation and evolution of the network. We summarized the enriched terms and unique enriched terms in Table 3. Besides finding the overrepresentation of protein counts in functional categories, enrichments were also calculated for functional categories for groups of proteins having significantly higher or lower values of network properties. For more details, see Additional file 1, Table S3 and S4.

**Table 3:** Summary of function enrichment tests

Temporal Group	Biological Process		Molecular Function	
	
	Significant Terms	Unique Terms	Significant Terms	Unique Terms
1	7	2	5	2
2	9	4	5	2
3	1	0	0	0
4	3	2	3	2
5	7	5	6	3
6	3	3	3	1

**Figure 4:**
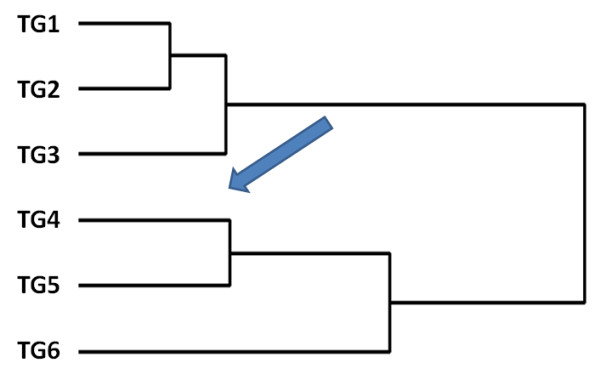
**A dendogram reflecting the functional relationships for proteins from different temporal groups**. The tree suggests a progressive functional change of the human PPI network during evolution. The observed clustering would not have occurred by random chance (P < 0.001). As indicated by the arrow, TG3 and TG4 in the tree are separately grouped into two clades, which are compared to the accelerated topology changes during this period as shown in Figure 3. TG: Temporal group.

We next trace the functional evolution of a PPI network, which has not been clearly investigated by previous studies. If the functional evolution of the network is adaptive, a progressive change in functions along with evolutionary age is hypothesized. By contrast, this is not expected if the change in function occurs at a single point in time or is neutral. To test this hypothesis, we first defined functional distance using the Mahalanobis distance between a pair of genes based on their function annotations (For details, see Methods). Compared to the functional similarity measurements used by other studies [27,28,30], the functional distance method used here considers the overall annotation pattern and is more informative. We calculated the functional distance for all possible pairs of nodes in the network, and the distances were further averaged and grouped into a 6 X 6 matrix using 6 X 6 TG combinations as indices (Table 4). By reordering the 6 X 6 matrix using agglomerative hierarchical clustering [34], as shown in Figure 4, the pattern of the tree obtained is consistent with the evolutionary time-scale, and with adjacent TGs being clustered with shorter functional distances (or higher functional similarity). To test whether the observed clustering is statistically significant, we calculate a p-value by comparing the observed summed distances along the tree against a null distribution produced by randomly permuting TG information of each protein (1000 permutations). In an attempt to quantify the relationship, we correlated the averaged functional distance with differences in group age, which has a coefficient of 0.725 (Spearman rho, p < 0.001), confirming a progressive functional change of the network. Interestingly, TG3 and TG4 in the tree are grouped separately into two clades. Considering our observation of accelerated topological changes during this period, this further indicates an association between topological evolution and functional evolution.

**Table 4:** Average functional distances across temporal groups

	TG1	TG2	TG3	TG4	TG5	TG6
TG1	4.413	4.560	5.344	6.109	6.512	6.018
TG2		4.321	5.054	5.643	6.070	6.311
TG3			4.996	5.900	6.189	5.595
TG4				5.921	5.938	4.967
TG5					4.903	4.377
TG6						2.975

The functional distance is the Mahalanobis distance between a pair of nodes (genes) based on their function annotations (for details, see Methods)

Distances are averaged according to the temporal group combinations to which each pair of nodes belongs.

TG: Temporal group

**Figure 5:**
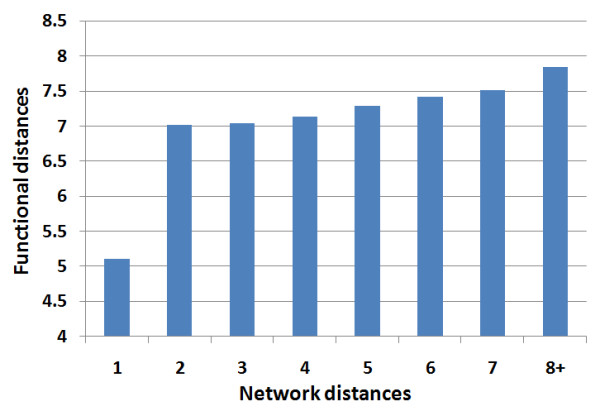
**Relationship between network distances and functional distances of the PPI network**. Network distances of eith and above were grouped. Mean functional distances were plotted for each network distance.

To what extent is network topology and function related? Pandey recently reported correlation between functional similarity and topological proximity [27]. Using our method, we detected statistically significant, but weak, correlation between functional distance and network distance (Spearman rho = 0.036, p < 0.001). When both distance measurements are grouped into the TG categories, the distance between TGs based on network distance and the distance between TGs based on functional distance are not correlated. This suggests that the correlation between functional distance and network distance is not universal. In more detail, we plot functional distance against network distance. As shown in Figure 5, although the directly interacting proteins show significantly shorter functional distances, there are only slight differences in functional distance for network distances greater than two. These observations suggest that function might not efficiently contribute to global organization of the network but instead might primarily contribute to the local organization during evolution. Since the local clustering coefficient measures the local organization of the network, we further hypothesize that the observed differences in clustering coefficients among TGs are mainly due to the differences in TG function. If this holds true, after gene function is controlled for, observed differences in clustering coefficients among TGs are expected to be reduced or even to disappear. We tested the hypothesis by comparing the global correlation coefficient with those obtained under each functional category individually. We found that the correlation between clustering coefficients and protein age significantly decreased after controlling for functional categories, with an after-control-value of -0.004 (Table 5). On the contrary, for both the interaction degree and network distance, we observed weaker correlations after controlling for functional categories, but found no significant differences (Table 5). Hence, although there is statistical enrichment of functional categories detected for interaction degree and network distance, function appears to not be the single determining factor affecting these two properties. Function does seem to be a dominant factor, however, contributing to the evolution of local clustering and topological modularity of the network.

**Table 5:** Changes in the correlation coefficients before and after functions are controlled for

	Interaction Degree		Clustering Coefficient		Network Distance	
	**Spearman correlation coefficient**	**Test statistics**	**Spearman correlation coefficient**	**Test statistics**	**Spearman correlation coefficient**	**Test statistics**

Intact	-0.212		-0.110		0.224	

Controlling Biological Process	-0.172	t = 1.98 df = 29 p = 0.058	-0.004	t = 2.95 df = 29 **p = 0.006**	0.191	t = -1.21 df = 29 p = 0.234

Controlling Molecular Function	-0.191	t = 1.06 df = 26 p = 0.299	-0.060	t = 2.29 df = 26 **p = 0.031**	0.209	t = -0.71 df = 26 p = 0.484

The Spearman correlation coefficient represents the tendency of network topologies to change along with temporal groups

All together, we show that PPI networks are functionally organized and under progressive functional evolution. Function might also substantially contribute to local clustering and topological modularity of these networks.

### The topological unit is also the functional unit

Based on the above findings, to further detect the association between functional and local topological organization of the network, we designed a new functional analysis, motivated by the concept of the clustering coefficient that measures a node's neighborhood density. We first defined a topological unit as a hub protein and all of its interacting partners in the network. We expect that members in the topological unit share a higher degree of functional similarity than do random nodes. Random networks were constructed with the same degree distribution but with randomly shuffled interaction partners. Taking into account the overall function changes for nodes from different TGs (as shown above), we tested our hypothesis by calculating a group distance, which is the average of all functional distances for partner pairs from different TGs for one single node. This approach will maximize the group distance to control the temporal effect (*i.e.*, genes in the same temporal group are functionally close, as shown above) in the empirical network, thus making the analysis more convincing. We found that the group distances for partners of hubs (we defined a hub with minimum degree of 50, n = 678) in the empirical network were significantly smaller than the values obtained from random networks (Kruskal-Wallis test, H = 62.69; df = 1; P < 0.0001), suggesting that partners are actually more functionally similar. Varying the definition of a hub to a minimum degree of 100 (n = 309) did not change the significance (Kruskal-Wallis test, H = 218.13; df = 1; P < 0.0001).

There are several considerations that might bias our result. The first consideration is that directly interacting proteins are functionally similar. Our PPI data show that interacting partners are more functionally similar (Kruskal-Wallis test, H = 53326.61; df = 7; P < 0.0001), which is consistent with previous reports [18,28]. We thus repeated the analysis by excluding neighbor pairs that are indeed interacting. The result was still statistically significant (Kruskal-Wallis test, H = 211.65; df = 1; P < 0.0001). The second consideration is duplication of interaction partners, because gene duplication plays a major role in evolution by providing material for evolution. Although it has been reported that 1), most duplicated genes experience a brief period of relaxed selection early in their history, many of them diverge significantly or are wiped out by natural selection due to accumulation of deleterious mutations [35]; and 2), only the most conserved pairs will retain their interaction [36]. However, some of the duplicated genes as well as the inherited interaction survive. This is the key point of the duplication-divergence model. If a hub protein X interacts with partner protein Y and Y', but Y' is duplicated from Y, Y and Y' are likely to have functional similarity due to the duplication. In order to control this situation, all human genes were clustered based on nucleotide sequence similarity. In brief, if gene A shows enough sequence similarity to gene B, and gene B shows enough sequence similarity to gene C, even if gene A is not similar to gene C, genes A, B and C will be put into one cluster. Clusters containing two or more genes thus show evidence of historic and detectable duplications. We first used the sequence similarity threshold of e^-25^. We excluded all genes from clusters containing two or more genes from our interaction data and repeated the analysis. After the correction, our results remained statistically significant (Kruskal-Wallis test, H = 201.08; df = 1; P < 0.0001). If we use loosened thresholds such as e^-10 ^or e^-20^, the results are quite similar (data not shown). Finally, since the interaction partners of a hub protein (that are not interacting with each other) are actually at a network distance of two, we also tested to see if the functional distance of interaction partners of a hub protein is smaller than the overall functional distance of proteins in the network with a network distance of two. Once again the result is consistent with what we expect: Members in the topological unit are more functionally condensed (Z = -68.79; P < 0.0001).

All in all, here we show topological modularity of the network as well as the functional modularity. The topological unit is also the functional unit of the network.

## Discussion

Current studies on the evolution of the PPI network focused mostly on topological properties, especially the cause of the power law degree distribution. A number of network models have been proposed. Preferential attachment is widely acknowledged as a candidate mechanism of generating a power law degree distribution for many networks, including the Web, publication citations and others. When the preferential attachment model is applied to PPI network evolution, it predicts that the interaction gain of a protein in the network is related to its connectivity at present. By adding a "fitness" parameter, Bianconi and Barabasi proposed an improved preferential attachment model called the "Fitness model," which gives the opportunity for latecomers to compete with existing nodes [37]. Some of our observations agree with what preferential attachment models predict. Since this model is not very biologically relevant and not able to capture the modularity in the empirical PPI network, it cannot be used to model the evolution of the PPI network.

Compared to the preferential attachment model, the duplication-divergence model may be more promising. It is more biologically plausible, and the network produced by the duplication-divergence model satisfies both the power law degree distribution and the modularity structure. However, duplication-divergence models are still derived more from a topological perspective. It is obvious that the evolution of the network is based on the evolution of proteins in the network. Unfortunately principles of molecular evolution are still largely ignored in the current duplication-divergence models. These models claim that both the observed degree distribution and the topological modularity of the network could be produced by gene duplication regardless of biological function and natural selection [7,13,30,38]. In the real world, every surviving gene and its interactions contribute to the organism's fitness according to its functional significance [39]. The fitness varies across specific biological functions and through stages of evolution. A gene or interaction with high fitness will survive in the next round of selection. Those interactions and the gene itself with low fitness will be selected against. The fitness of modularity is less studied because modularity does not interact with the environment directly, thus it was thought that it might not contribute to the fitness of an organism. However, simulation studies suggest modularity would directly benefit fitness by providing evolvability [40]. Modularity can also contribute to the fitness of an organism by increasing "error tolerance" through limiting the contributions of the fitness of genes in the module [41]. We found a connection between topological modularity and functional modularity by showing that the topological unit is also the functional unit. The topological modularity appears to carry functional information and less likely to be a pure byproduct of stochastic processes. So is the evolution of the overall network.

## Conclusions

In this study, we show that the human PPI network is functionally organized and evolving. The evolution of function is consistent with the evolution of network topologies. Function might substantiality contribute to the local topological modularity of a PPI network. Although the functional evolution is hard to incorporate into current stochastic models, we suggest that it cannot be simply ignored when studying PPI network evolution.

## Methods

### Protein interaction and annotation database

Nucleotide sequences used in this study were collected from two sources: the NCBI Reference Sequence (RefSeq) database for human and mouse ftp://ftp.ncbi.nih.gov/refseq/[42] and the Unigene database for all other species as listed below ftp://ftp.ncbi.nih.gov/repository/UniGene/[43]. Human protein-protein interaction data was integrated from three sources: BioGrid http://thebiogrid.org[44], HPRD http://www.hprd.org[45] and REACTOME http://www.reactome.org[46]. Functional annotations for human genes were retrieved from the PANTHER database ftp://ftp.pantherdb.org[47] and the GO database http://geneontology.org[48].

### Human gene temporal group construction

All human genes were classified into six temporal groups based on a nucleotide sequence similarity search using BLAST [49] against several clades in the known evolutionary tree [33] with an E-value threshold set to e^-20^. In detail, if a human gene has a homolog in either the fruitfly, mosquito, nematode or schistosoma species with the nucleotide similarity over the threshold, it was classified into the oldest temporal group. Similarly, if a second gene has a homolog in either the pufferfish, medaka, trout or zebrafish species but not in the first clade, the assumption was made that it was introduced at this stage in the phylogenetic chain and was therefore placed in the second temporal group. The species used for each temporal group are: TG1 (African malaria mosquito, Fruitfly, Nematode, Schistosoma and Yellow fever mosquito), TG2 (Medaka, Pufferfish, Trout and Zebrafish), TG3 (Clawed frog and Tropical frog), TG4 (Chicken), TG5 (Cattle, Dog, Pig and Sheep), and TG6 (all human genes not found in the other species). See Additional file 1, Figure S1 for more details. Since the distribution of genes among the six different temporal groups would be sensitive to the threshold E-value used to allocate genes. We also tried a looser threshold e^-10 ^and a stricter threshold e^-30 ^for the classifying. Our conclusion was basically not affected by which threshold was chose. We thus report the results using the threshold of e^-20^, which is more commonly used by other studies. Results using other thresholds are provided in Additional file 1, Table S5 and S6.

### Interaction density

The interaction density between two temporal groups was calculated as [14]:

$$D_{m,n}=I_{m,n}/E_{m,n}$$

$$E_{m,n}=N_{m}\times N_{n}\;\left(m\neq n\right)$$

$$E_{m,n}=N_{m}\left(N_{n}-1\right)/2\;\left(m=n\right)$$

Where *I*m, n and *E*m, n are the observed and all possible interactions between temporal group m and n in the PPI network, respectively. N is the number of proteins that are in the PPI network of a particular temporal group.

### Ka/Ks

Coding sequences (CDS) of human and mouse were extracted from RefSeq transcripts. Orthologs between human and mouse were identified using reciprocal blast with the threshold of e^-50^. Orthologous protein pairs were aligned using ClustalW and then back translated into a nucleotide sequence alignment. For a nucleotide sequence, Ka is defined as the number of nonsynonymous substitutions per nonsynonymous site and Ks as the number of synonymous substitutions per synonymous site. Ka/Ks (Omega) is the index of strength of selective constraint. Ka and Ks are estimated using the maximum likelihood method implemented in the codeml program under the F3 } 4 model of codon substitution [50].

### Gene functional distance

We used two different methods to calculate the gene functional distance. For a direct method, genes were first represented in vector space, where each vector denoted presence or absence of a functional term. If there is an annotation for this functional term, that term's position in the vector is set to 1, otherwise it is set to 0. Considering the hierarchical structure of function annotations, we used only the sub-root level annotations (the direct children of biological process/molecular function) for each transcript to avoid redundancy. Functional distance is calculated as the Mahalanobis distance, measured for the vectors. Mahalanobis distance was used because it considers the dependence of the annotation terms, which is reflected in the covariance matrix. We also used a two-step semantic similarity based method implemented by R package csbl.go [51]. In this method, the semantic similarity between each pair of GO annotation terms was first computed according to Resnik [52] and gene functional similarity was then measured by the maximum of pair wise term similarities for the gene pair [53]. The gene similarities were finally 1/2^x ^transformed into distance-like measures.

Using either method, or using the annotation "Biological Process" or "Molecular Function", will not affect our conclusion. We thus reported functional distances calculated from "Biological Process" annotations from Mahalanobis distance method only in this manuscript. Gene functional distance data from "Biological Process" and "Molecular Function" of both methods can be downloaded at http://www.mooneygroup.org/yiqiang/PPI_data/.

### Statistical tests

We used the Kruskal-Wallis test for comparing populations in this study. Kruskal-Wallis test is non-parametric one-way analysis of variance which does not assume that the data are normally distributed. Kruskal-Wallis test is an extension of the Mann-Whitney U test to three or more groups and it is equivalent to the Mann-Whitney U test when applying for two groups. Function enrichment/overrepresentation of specific functional annotations was determined by the hypergeometric test. The z-score was used to measure if proteins in some functional categories had significantly higher or lower network properties. The statistical significance was then accessed according to the Gaussian distribution. Considering the hierarchical structure of function annotations, we used only the sub-root level annotation (the annotation just under biological process and molecular function) for each gene to do the function enrichment test. P-values are corrected by the Benjamini-Hochberg (BH) method.

## Competing interests

The authors declare that they have no competing interests.

## Authors' contributions

YZ and SDM designed the experiments and drafted the manuscript. Both authors read and approved the manuscript.

## Supplementary Material

Additional file 1 **A file containing additional data: 1 additional figure, 6 additional tables**. Click here for file
