# Supplementary material for: Functional organization and its implication in evolution of the human protein-protein interaction network
Source: BMC Genomics. 2012 Apr 24;13:150. doi: 10.1186/1471-2164-13-150 (PMC3375200; doi:10.1186/1471-2164-13-150)
Supplement: Additional file 1 — A file containing additional data: 1 additional figure, 6 additional tables. [file 1471-2164-13-150-S1.DOC]

Additional Figure S1. Human gene temporal group construction.


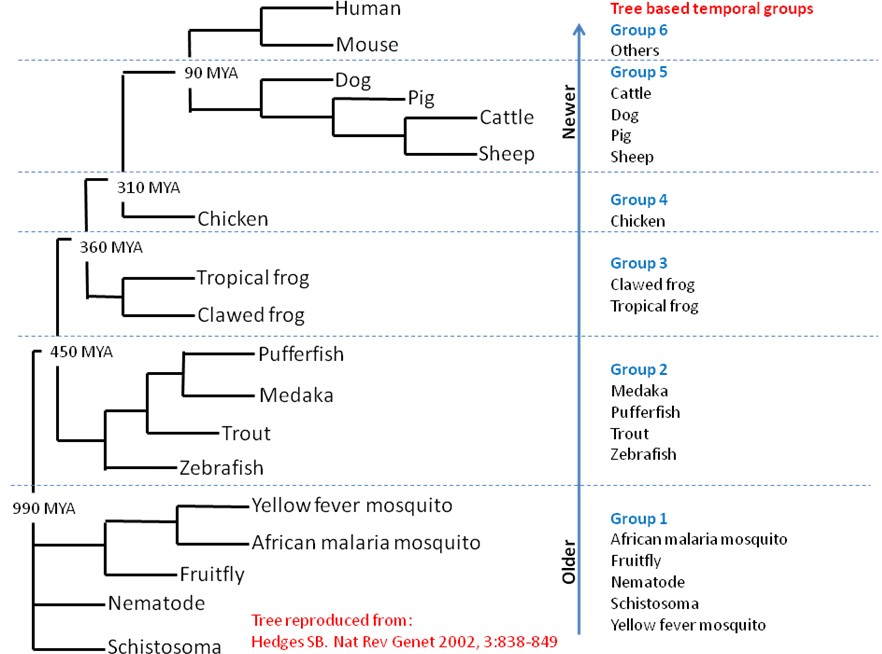


Branch lengths of the tree are not proportional to the evolutionary distances

Additional Table S1. Interaction densities across temporal groups, not normalized.

|  | TG1 | TG2 | TG3 | TG4 | TG5 | TG6 |
| --- | --- | --- | --- | --- | --- | --- |
| TG1 | 4.118E-03 | 2.233E-03 | 1.244E-03 | 7.630E-04 | 4.755E-04 | 5.818E-05 |
| TG2 |  | 1.189E-03 | 6.643E-04 | 4.173E-04 | 2.393E-04 | 2.938E-05 |
| TG3 |  |  | 5.050E-04 | 3.019E-04 | 1.863E-04 | 2.803E-05 |
| TG4 |  |  |  | 2.211E-04 | 1.659E-04 | 2.064E-05 |
| TG5 |  |  |  |  | 1.776E-04 | 2.835E-05 |
| TG6 |  |  |  |  |  | 3.251E-05 |

Additional Table S2. Rate of change of the network properties, not normalized.

|  | TG1-TG2 | TG2-TG3 | TG3-TG4 | TG4-TG5 |
| --- | --- | --- | --- | --- |
| Network Distance | 2.765E-04 | 6.761E-04 | 1.788E-03 | 4.318E-04 |
| Interaction Degree | 2.243E-02 | 4.777E-02 | 6.078E-02 | 6.535E-03 |
| Clustering Coefficient | 2.989E-05 | 3.053E-05 | 2.624E-04 | 4.822E-05 |

Additional Table S3. Function overrepresentation test for proteins of each temporal group in the PPI network.

| Temporal Group | Function Category | Annotation | N1 | N2 | P value* |
| --- | --- | --- | --- | --- | --- |
| 1 | BP00031 | Nucleoside, nucleotide and nucleic acid metabolism | 3237 | 269 | 0.000E+00 |
| 1 | BP00203 | Cell cycle | 941 | 89 | 4.977E-10 |
| 1 | BP00125 | Intracellular protein traffic | 946 | 83 | 6.306E-08 |
| 1 | BP00193 | Developmental processes | 1972 | 143 | 1.066E-07 |
| 1 | BP00285 | Cell structure and motility | 1118 | 90 | 7.079E-07 |
| 1 | BP00173 | Muscle contraction | 191 | 23 | 4.157E-05 |
| 1 | BP00267 | Homeostasis | 187 | 21 | 2.277E-04 |
| 2 | BP00166 | Neuronal activities | 548 | 281 | 0.000E+00 |
| 2 | BP00193 | Developmental processes | 1972 | 759 | 0.000E+00 |
| 2 | BP00125 | Intracellular protein traffic | 946 | 383 | 1.351E-12 |
| 2 | BP00141 | Transport | 1276 | 477 | 2.348E-09 |
| 2 | BP00031 | Nucleoside, nucleotide and nucleic acid metabolism | 3237 | 1077 | 1.099E-06 |
| 2 | BP00203 | Cell cycle | 941 | 330 | 3.194E-04 |
| 2 | BP00060 | Protein metabolism and modification | 2973 | 961 | 6.045E-04 |
| 2 | BP00285 | Cell structure and motility | 1118 | 384 | 6.074E-04 |
| 2 | BP00095 | Phosphate metabolism | 114 | 51 | 6.767E-04 |
| 3 | BP00289 | Other metabolism | 507 | 72 | 2.353E-04 |
| 4 | BP00124 | Cell adhesion | 607 | 129 | 1.380E-10 |
| 4 | BP00101 | Sulfur metabolism | 93 | 24 | 2.277E-04 |
| 4 | BP00019 | Lipid, fatty acid and steroid metabolism | 746 | 123 | 2.397E-04 |
| 5 | BP00148 | Immunity and defense | 1271 | 641 | 0.000E+00 |
| 5 | BP00019 | Lipid, fatty acid and steroid metabolism | 746 | 279 | 1.823E-09 |
| 5 | BP00076 | Electron transport | 243 | 108 | 1.859E-08 |
| 5 | BP00209 | Blood circulation and gas exchange | 87 | 48 | 7.588E-08 |
| 5 | BP00289 | Other metabolism | 507 | 187 | 2.867E-06 |
| 5 | BP00179 | Apoptosis | 491 | 179 | 9.868E-06 |
| 5 | BP00224 | Cell proliferation and differentiation | 986 | 322 | 1.392E-04 |
| 6 | BP00102 | Signal transduction | 3325 | 246 | 0.000E+00 |
| 6 | BP00182 | Sensory perception | 495 | 154 | 0.000E+00 |
| 6 | BP00281 | Oncogenesis | 396 | 34 | 1.960E-05 |
|  |  |  |  |  |  |
| 1 | MF00042 | Nucleic acid binding | 2692 | 212 | 1.605E-15 |
| 1 | MF00036 | Transcription factor | 1781 | 142 | 2.584E-10 |
| 1 | MF00091 | Cytoskeletal protein | 816 | 70 | 1.519E-06 |
| 1 | MF00107 | Kinase | 661 | 57 | 1.292E-05 |
| 1 | MF00024 | Ion channel | 337 | 32 | 1.885E-04 |
| 2 | MF00024 | Ion channel | 337 | 179 | 0.000E+00 |
| 2 | MF00107 | Kinase | 661 | 337 | 0.000E+00 |
| 2 | MF00036 | Transcription factor | 1781 | 624 | 5.108E-07 |
| 2 | MF00093 | Select regulatory molecule | 1082 | 385 | 2.538E-05 |
| 2 | MF00267 | Membrane traffic protein | 344 | 134 | 2.842E-04 |
| 4 | MF00040 | Cell adhesion molecule | 389 | 105 | 1.605E-15 |
| 4 | MF00178 | Extracellular matrix | 329 | 72 | 6.083E-07 |
| 4 | MF00082 | Transporter | 636 | 112 | 3.419E-05 |
| 5 | MF00001 | Receptor | 1392 | 532 | 0.000E+00 |
| 5 | MF00016 | Signaling molecule | 760 | 335 | 0.000E+00 |
| 5 | MF00173 | Defense/immunity protein | 350 | 237 | 0.000E+00 |
| 5 | MF00123 | Oxidoreductase | 583 | 241 | 2.390E-13 |
| 5 | MF00153 | Protease | 552 | 203 | 1.326E-06 |
| 5 | MF00178 | Extracellular matrix | 329 | 119 | 4.511E-04 |
| 6 | MF00001 | Receptor | 1392 | 212 | 0.000E+00 |
| 6 | MF00190 | Viral protein | 4 | 3 | 1.031E-05 |
| 6 | MF00173 | Defense/immunity protein | 350 | 31 | 2.504E-05 |

N1: Gene counts in the human genome

N2: Gene counts in the human PPI network

BP: Biological process

MF: Molecular function

* P value after BH correction

Additional Table S4. Function Z-score test for proteins of each temporal group in the PPI network.

| Network Property | Temporal Group | Function Category | Annotation | Z-score | P value* |
| --- | --- | --- | --- | --- | --- |
| Interaction Degree | 1 | BP00060 | Protein metabolism and modification | 11.521 | 0.000E+00 |
|  | 1 | BP00102 | Signal transduction | 8.922 | 0.000E+00 |
|  | 1 | BP00031 | Nucleoside, nucleotide and nucleic acid metabolism | 6.165 | 9.778E-09 |
|  | 1 | BP00203 | Cell cycle | 5.887 | 4.660E-08 |
|  | 1 | BP00285 | Cell structure and motility | 5.427 | 5.942E-07 |
|  | 1 | BP00137 | Protein targeting and localization | 5.102 | 2.541E-06 |
|  | 1 | BP00224 | Cell proliferation and differentiation | 3.968 | 3.005E-04 |
|  | 1 | BP00179 | Apoptosis | 3.980 | 3.005E-04 |
|  | 1 | BP00281 | Oncogenesis | 3.870 | 4.302E-04 |
|  | 2 | BP00060 | Protein metabolism and modification | 9.021 | 0.000E+00 |
|  | 2 | BP00281 | Oncogenesis | 6.495 | 1.727E-09 |
|  | 2 | BP00031 | Nucleoside, nucleotide and nucleic acid metabolism | 5.111 | 2.541E-06 |
|  | 2 | BP00224 | Cell proliferation and differentiation | 5.074 | 2.692E-06 |
|  | 2 | BP00203 | Cell cycle | 4.813 | 9.472E-06 |
|  | 2 | BP00102 | Signal transduction | 4.629 | 2.179E-05 |
|  | 2 | BP00179 | Apoptosis | 3.800 | 5.464E-04 |
|  | 5 | BP00076 | Electron transport | 4.369 | 6.103E-05 |
|  |  |  |  |  |  |
|  | 1 | MF00042 | Nucleic acid binding | 12.631 | 0.000E+00 |
|  | 1 | MF00131 | Transferase | 7.328 | 5.929E-12 |
|  | 1 | MF00093 | Select regulatory molecule | 5.975 | 4.366E-08 |
|  | 1 | MF00107 | Kinase | 5.829 | 7.079E-08 |
|  | 1 | MF00077 | Chaperone | 4.875 | 9.197E-06 |
|  | 2 | MF00042 | Nucleic acid binding | 16.545 | 0.000E+00 |
|  | 2 | MF00093 | Select regulatory molecule | 5.899 | 5.550E-08 |
|  | 3 | MF00042 | Nucleic acid binding | 4.678 | 2.204E-05 |
|  |  |  |  |  |  |
| Clustering Coefficient | 1 | BP00060 | Protein metabolism and modification | 4.792 | 3.425E-05 |
|  | 1 | BP00076 | Electron transport | 4.267 | 3.284E-04 |
|  | 2 | BP00031 | Nucleoside, nucleotide and nucleic acid metabolism | 5.016 | 1.461E-05 |
|  | 2 | BP00060 | Protein metabolism and modification | 5.061 | 1.461E-05 |
|  | 3 | BP00031 | Nucleoside, nucleotide and nucleic acid metabolism | 4.119 | 5.254E-04 |
|  | 5 | BP00076 | Electron transport | 8.174 | 3.686E-14 |
|  |  |  |  |  |  |
|  | 1 | MF00042 | Nucleic acid binding | 5.407 | 2.429E-06 |
|  | 2 | MF00042 | Nucleic acid binding | 10.021 | 0.000E+00 |
|  | 3 | MF00042 | Nucleic acid binding | 4.359 | 3.307E-04 |
|  | 4 | MF00042 | Nucleic acid binding | 3.972 | 9.020E-04 |
|  | 5 | MF00123 | Oxidoreductase | 4.072 | 7.093E-04 |
|  |  |  |  |  |  |
| Network Distance | 1 | BP00060 | Protein metabolism and modification | -7.969 | 6.651E-14 |
|  | 1 | BP00102 | Signal transduction | -7.780 | 2.004E-13 |
|  | 1 | BP00203 | Cell cycle | -5.458 | 2.103E-07 |
|  | 1 | BP00285 | Cell structure and motility | -4.863 | 4.232E-06 |
|  | 1 | BP00224 | Cell proliferation and differentiation | -4.850 | 4.268E-06 |
|  | 1 | BP00281 | Oncogenesis | -3.960 | 1.882E-04 |
|  | 2 | BP00224 | Cell proliferation and differentiation | -7.278 | 7.027E-12 |
|  | 2 | BP00060 | Protein metabolism and modification | -6.271 | 4.249E-09 |
|  | 2 | BP00203 | Cell cycle | -5.932 | 3.111E-08 |
|  | 2 | BP00031 | Nucleoside, nucleotide and nucleic acid metabolism | -5.757 | 5.922E-08 |
|  | 2 | BP00281 | Oncogenesis | -5.691 | 8.077E-08 |
|  | 2 | BP00102 | Signal transduction | -5.465 | 2.103E-07 |
|  | 2 | BP00179 | Apoptosis | -5.467 | 2.103E-07 |
|  | 2 | BP00148 | Immunity and defense | -3.719 | 4.362E-04 |
|  | 2 | BP00285 | Cell structure and motility | -3.652 | 5.398E-04 |
|  | 3 | BP00203 | Cell cycle | -3.865 | 2.630E-04 |
|  |  |  |  |  |  |
|  | 1 | MF00107 | Kinase | -6.466 | 1.274E-09 |
|  | 1 | MF00093 | Select regulatory molecule | -4.147 | 1.278E-04 |
|  | 1 | MF00042 | Nucleic acid binding | -3.921 | 2.680E-04 |
|  | 1 | MF00077 | Chaperone | -3.846 | 3.375E-04 |
|  | 2 | MF00042 | Nucleic acid binding | -8.677 | 3.088E-16 |
|  | 2 | MF00107 | Kinase | -7.591 | 8.049E-13 |
|  | 2 | MF00036 | Transcription factor | -6.469 | 1.274E-09 |
|  | 2 | MF00093 | Select regulatory molecule | -5.919 | 2.740E-08 |
|  | 2 | MF00197 | Miscellaneous function | -3.595 | 7.953E-04 |
|  | 3 | MF00042 | Nucleic acid binding | -4.065 | 1.606E-04 |
|  | 3 | MF00036 | Transcription factor | -3.854 | 3.375E-04 |

BP: Biological process

MF: Molecular function

* P value after BH correction

Additional Table S5. Properties of the PPI network for each temporal group using the BLAST threshold of e^-10^.

| Temporal group | Gene number in the interaction network | Average interaction degree | Average clustering coefficient | Network Distance | Average Omega |
| --- | --- | --- | --- | --- | --- |
| 1 | 1073 | 25.05871 | 0.199777 | 3.912931 | 0.041405 |
| 2 | 4309 | 15.52564 | 0.191256 | 4.064536 | 0.097544 |
| 3 | 951 | 11.08517 | 0.187084 | 4.140065 | 0.123466 |
| 4 | 985 | 9.088325 | 0.171148 | 4.242504 | 0.150774 |
| 5 | 2073 | 8.049686 | 0.174547 | 4.278879 | 0.19556 |
| 6 | 139 | 3.28777 | 0.194108 | 4.459318 | 0.179563 |

Additional Table S6. Properties of the PPI network for each temporal group using the BLAST threshold of e^-30^.

| Temporal group | Gene number in the interaction network | Average interaction degree | Average clustering coefficient | Network Distance | Average Omega |
| --- | --- | --- | --- | --- | --- |
| 1 | 322 | 28.06211 | 0.197073 | 3.9149 | 0.02805 |
| 2 | 2893 | 19.49844 | 0.196911 | 3.982411 | 0.088273 |
| 3 | 1150 | 14.91913 | 0.20695 | 4.065278 | 0.082324 |
| 4 | 1562 | 10.6767 | 0.185373 | 4.159905 | 0.110872 |
| 5 | 3318 | 9.005726 | 0.168427 | 4.253408 | 0.167981 |
| 6 | 285 | 4.442105 | 0.190505 | 4.394404 | 0.194 |
